# Composite Metagenome-Assembled Genomes Reduce the Quality of Public Genome Repositories

**DOI:** 10.1128/mBio.00725-19

**Published:** 2019-06-04

**Authors:** Alon Shaiber, A. Murat Eren

**Affiliations:** a Graduate Program in Biophysical Sciences, University of Chicago, Chicago, Illinois, USA; b Department of Medicine, University of Chicago, Chicago, Illinois, USA; c Josephine Bay Paul Center, Marine Biological Laboratory, Woods Hole, Massachusetts, USA; Stanford University

## LETTER

In their recent study, Espinoza et al. employ genome-resolved metagenomics to investigate supragingival plaque metagenomes of 88 individuals (1). The 34 metagenome-assembled genomes (MAGs) that the authors report include those that resolve to clades that have largely evaded cultivation efforts, such as *Gracilibacteria* (formerly GN02) and *Saccharibacteria* (formerly TM7) of the recently described Candidate Phyla Radiation (2). Generating new genomic insights into the understudied members of the human oral cavity is of critical importance for a comprehensive understanding of the microbial ecology and functioning of this biome, and we acknowledge the contribution of the authors on this front. However, the redundant occurrence of bacterial single-copy core genes suggests that more than half of the MAGs that Espinoza et al. report are composite genomes that do not meet the recent quality guidelines suggested by the community (3). Composite genomes that aggregate sequences originating from multiple distinct populations can yield misleading insights when treated and reported as single genomes (4).

**FIG 1:**
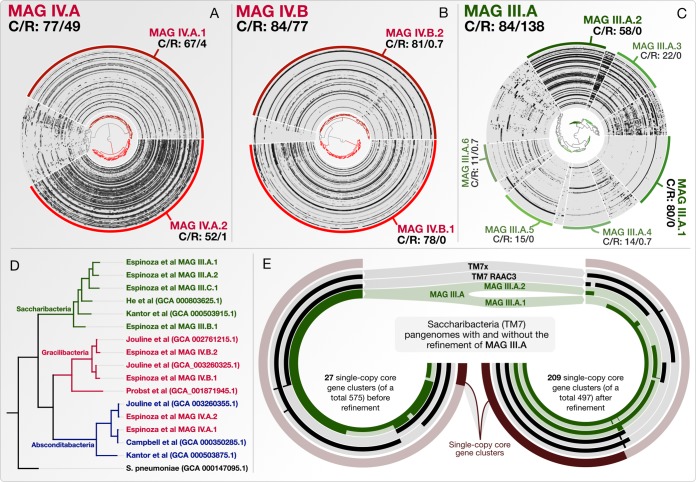
Refinement of three composite genome bins. (A to C) The top left corners of these panels display the original name of a given Espinoza et al. MAG (see Table 1 in the original study) and its estimated completion and redundancy (C/R) based on a bacterial single-copy core gene collection (10). Each concentric circle represents one of the 88 metagenomes in the original study, dendrograms show hierarchical clustering of contigs based on sequence composition and differential mean coverage across metagenomes (using Euclidean distance and Ward’s method), and each data point represents the read recruitment statistic of a given contig in a given metagenome. Arcs at the outermost layers mark contigs that belong to a refined bin along with their new completion and redundancy estimates (C/R). (D) The phylogenomic tree organizes genomes based on 37 concatenated ribosomal proteins. Coloring of genome names matches their taxonomy in NCBI, and branch colors match the consensus taxonomy of genomes they represent. Espinoza et al. reported MAG IV.A as *Gracilibacteria* (hence the red color); however, this phylogenomic analysis places refined MAGs under *Absconditabacteria*. (E) Pangenomic analysis of Espinoza et al. *Saccharibacteria* MAG III.A before (left) and after (right) refinement together with the *Saccharibacteria* genomes from panel D. Pangenomes describe 575 and 497 gene clusters, respectively, where each concentric circle represents a genome and bars correspond to the number of genes that a given genome is contributing to a given gene cluster (the maximum value is set to 2 for readability). Outermost layers mark single-copy core gene clusters to which every genome contributes precisely a single gene. We used Bowtie2 (11) to recruit reads from metagenomes, and anvi’o (12) to visualize and refine Espinoza et al. MAGs. FAMSA (13) aligned anvi’o-reported ribosomal protein amino acid sequences, trimAl (14) curated them, and IQ-TREE (15) computed the tree for the phylogenomic analysis. Anvi’o used DIAMOND (16) and MCL (17) algorithms to determine pangenomes. A reproducible bioinformatics workflow and FASTA files for refined MAGs are available at http://merenlab.org/data/refining-espinoza-mags.

To briefly demonstrate their composite nature, we refined some of the key Espinoza et al. MAGs through a previously described approach (5) and the data that the authors kindly provided (1). We found that MAG IV.A, MAG IV.B, and MAG III.A described multiple discrete populations with distinct distribution patterns across individuals (Fig. 1). A phylogenomic analysis of refined MAG IV.A genomes resolved to the candidate phylum *Absconditabacteria* (formerly SR1) and not to *Gracilibacteria* as reported by Espinoza et al. (Fig. 1D). A pangenomic analysis of the original and refined MAG III.A genomes with other publicly available *Saccharibacteria* genomes showed a 7-fold increase in the number of single-copy core genes (Fig. 1E). These findings demonstrate the potential implications of composite MAGs in comparative genomics studies where single-copy core genes are commonly used to infer diversity, phylogeny, and taxonomy (6). Composite MAGs can also lead to inaccurate ecological insights through inflated abundance and prevalence estimates. For instance, the original MAG III.A recruited a total of 1,849,593 reads from Espinoza et al. metagenomes; however, the most abundant refined III.A genome (MAG III.A.2, Fig. 1C) recruited only 629,291 reads.

Co-assembly of a large number of metagenomes that contain very closely related populations often hinders confident assignments of shared contigs into individual bins. Nevertheless, even when proper refinement is not possible, reporting composite MAGs as single genomes should be avoided. As of today, highly composite Espinoza et al. MAGs (Fig. 1 in this letter and Table 1 in the work of Espinoza et al.) are available as single genomes in public databases of the National Center for Biotechnology Information (NCBI).

The rapidly increasing number of MAGs in public databases already competes with the total number of microbial isolate genomes (3), and increasingly frequent studies that report large collections of MAGs offer a glimpse of the future (7–9). Despite their growing availability, metagenomes are inherently complex and demand researchers to orchestrate an intricate combination of rapidly evolving computational tools and approaches with many alternatives to reconstruct, characterize, and finalize MAGs. We must continue to champion studies such as the one by Espinoza et al. for their contribution to our collective effort to shed light on the darker branches of the ever-growing Tree of Life. At the same time, editors and reviewers of genome-resolved metagenomics studies should properly scrutinize the quality and accuracy of MAGs prior to their publication. A systematic failure at this will reduce the quality of public genome repositories while yielding adverse effects such as misleading insights into novel microbial groups and reduced trust among scientists in findings that emerge from genome-resolved metagenomics.
